# Innate Immune Responses and *P. falciparum* CS Repeat-Specific Neutralizing Antibodies Following Vaccination by Skin Scarification

**DOI:** 10.3389/fimmu.2022.801111

**Published:** 2022-06-06

**Authors:** Robert A. Mitchell, Rita Altszuler, Sandra Gonzalez, Roshawn Johnson, Ute Frevert, Elizabeth Nardin

**Affiliations:** Department of Microbiology, New York University School of Medicine, New York, NY, United States

**Keywords:** *Plasmodium falciparum*, circumsporozoite protein, peptide, skin scarification, Toll-Like Receptor (TLR) agonist adjuvants, innate immunity, IP-10 (CXCL-10), antibody

## Abstract

The skin is the site of host invasion by the mosquito-borne *Plasmodium* parasite, which caused an estimated 229 million infections and 409,000 deaths in 2019 according to WHO World Malaria report 2020. In our previous studies, we have shown that skin scarification (SS) with a *P. falciparum* circumsporozoite (CS) peptide in the oil-in-water adjuvant AddaVax containing a combination of TLR 7/8 and TLR 9 agonists can elicit sporozoite neutralizing antibodies. SS with AddaVax + TLR agonists, but not AddaVax alone, elicited CD4+ Th1 cells and IgG2a/c anti-repeat antibody. To explore the innate immune responses that may contribute to development of adaptive immunity following SS, we examined the skin at 4h and 24h post priming with CS peptide in AddaVax with or without TLR agonists. H&E stained and IHC-labeled dorsal skin sections obtained 24h post SS demonstrated a marked difference in the pattern of infiltration with F4/80+, CD11b+ and Ly6G+ cells at the immunization site, with the lowest intensity noted following SS with AddaVax + TLR agonists. Serum collected at 4h post SS, had reproducible increases in IL-6, MIP-3α, IL-22 and IP-10 (CXCL10) following SS with AddaVax + TLR agonists, but not with AddaVax alone. To begin to decipher the complex roles of these pro-inflammatory cytokines/chemokines, we utilized IP-10 deficient (IP-10 -/-) mice to examine the role of this chemokine in the development of anti-repeat antibody response following SS. In the absence of IP-10, the levels of Th1-type IgG2a/c antibody and kinetics of the primary anti-repeat antibody response were reduced following prime and boost. The IP-10 chemokine, present as early as 4h post prime, may provide an early serological marker for rapid screening of adjuvant formulations and delivery platforms to optimize SS-induced humoral immunity to CS repeats as well as other pathogens.

## Introduction

The skin provides the first barrier against pathogens that directly invade the host or that are delivered into the skin by the bite of arthropod vectors, as in the case of the *Plasmodium* parasite. Studies in rodents, non-human primates and human volunteers have shown that sterile immunity can be elicited by sporozoites delivered by the bite of *Plasmodium*-infected mosquitoes (1–6). While immunization by exposure to the bites of infected mosquitoes is not practical for mass vaccination campaigns, the analysis of immune responses in sporozoite-immunized hosts has provided critical information on the immune mechanisms that effectively target the infective sporozoite.

Antibody specific for the major surface antigen of the sporozoite, the circumsporozoite protein (CS), was one of the first protective immune mechanisms identified in sporozoite-immunized experimental hosts (7, 8). Murine monoclonal antibodies (MAB) that target the CS repeats, derived from sporozoite-immunized mice, were shown to neutralize sporozoite infectivity by inhibiting parasite motility in the skin and by blocking liver invasion thus preventing the subsequent development of Plasmodium blood stages responsible for clinical disease (9–11). In more recent studies, human CS-specific MAB derived from volunteers immunized with *P. falciparum* sporozoites were shown to protect human liver-chimeric mice against *P. falciparum* sporozoite challenge (12, 13).

A significant advance in malaria vaccine development has been a CS-based recombinant protein vaccine, termed RTS,S, that was shown in Phase III trials to protect 30-50% of immunized infants and children in Africa (14). RTS,S-induced protection was predominantly antibody-mediated (15, 16). Human MABs targeting CS repeats derived from the RTS,S vaccinees were shown to reduce *P. falciparum* sporozoite infectivity *in vitro* and infection of human liver chimeric mice (17, 18). RTS,S was recently recommended by the WHO for use in children living in moderate to high malaria transmission countries in Africa (19). Encouraged by these advances, efforts continue to improve CS-based vaccine efficacy and delivery methods.

The large scale deployment of vaccines in resource poor areas requires ease of administration by trained community workers, as was successfully used in the WHO Smallpox Eradication Campaign. In previous murine studies, we utilized a two-pronged stylet, as used for administration of smallpox vaccine, to immunize mice by skin scarification (SS) with a *P. falciparum* CS repeat peptide (20). Preclinical testing of highly purified CS-based subunit vaccines have illustrated the critical role of adjuvant in eliciting sporozoite neutralizing antibodies. Potent new adjuvants based on well defined synthetic TLR agonists that specifically target cellular receptors have been developed (21, 22). We therefore utilized adjuvants containing the TLR 7/8 agonist Resiquimod and the TLR 9 agonist CpG (20), as these bind to receptors within the endosome where co-localization with endocytosed antigen may more closely mimic the innate immune patterns elicited by infectious pathogens (23). Our prior SS studies (20) found that induction of anti-repeat antibodies that neutralized infectivity of sporozoites required a combination of TLR 7/8 and TLR 9 agonists in Addvax, a squalene-based oil-in-water nano-emulsion adjuvant.

The current studies were undertaken to explore early innate immune responses that play a role in development of anti-repeat antibody following SS with or without TLR agonists. We examined the SS site using H&E stained and IHC-labeled skin sections as well as measuring systemic cytokines/chemokines in serum collected at 4h and 24h post post prime. A better understanding of innate immune responses associated with the development of anti-repeat IgG antibodies will facilitate optimization of vaccine-induced humoral immunity to target the extracellular sporozoite and prevent development of clinical disease.

## Materials and Methods

### Immunization

C57Bl/6 mice 6-8 weeks of age and breeding pairs of IP-10 -/- mice (B6.129S4-Cxcl10^tm1Adl/^J) lacking IP-10 (CXCL10) were obtained from Jackson Laboratories (Bar Harbor, Maine). Mice were immunized by SS at 14-28 day intervals with one to four doses of a *P. falciparum* CS peptide delivered into the interscapular dorsal area using a two pronged stylet (Precision Medical Inc., Denver, PA), as previously described (20). The tetrabranched CS peptide used as antigen was comprised of tandem copies of both the major repeats (NANP)_3_ and the minor repeats (DPNANPNVDPNANPNV) that are contained in the CS repeat region (24, 25). The CS repeat peptide (50 µg) was administered either with or without TLR agonists in AddaVax (InvivoGen, San Diego, CA), a squalene oil-in-water nano-emulsion, comparable to MF59 adjuvant approved for human vaccines, which is known to function as an antigen depot and enhance Th2-type antibody responses (26). A total volume of 100 - 200 µl of vaccine formulation was applied to a 2 cm^2^ area of unshaved dorsal skin followed by 10 pricks with the two-pronged stylet. Innate immune responses were measured in serum and skin sections obtained at 4h and 24h after SS priming. For immunogenicity experiments, a prime-boost immunization schedule was performed where two prime doses were administered followed by two additional booster doses. Humoral immunity was measured using serum obtained 14-28 days after each of four SS immunizations delivered at 14 – 28 day intervals. T cell responses were measured using spleen cells obtained after the final immunization. The study was conducted in strict accordance with the recommendations in the Guide for the Care and Use of Laboratory Animals of the National Institutes of Health. The protocol was approved by the Institutional Animal Care and Use Committee, NYU School of Medicine.

### TLR Agonists

The TLR agonists used in the adjuvant formulation included the TLR 7/8 agonist Resiquimod (*In vivo*Gen, San Diego, CA), an imidazoquinoline derivative with well-defined adjuvant properties in murine and human hosts (22, 27). The TLR 9 agonist used was CpG, a cytosine:guanine oligodeoxyribonucleotide (ODN) that mimics an unmethylated bacterial DNA motif synthesized on a phosphorothioate backbone (The Certified Midland Reagent Co., Midland, TX) (28). The TLR agonists were tested using the manufacturer’s recommended dose per mouse (range 125-150 µg/dose).

### Histology and Immunohistochemical (IHC) Staining

**Figure 1:**
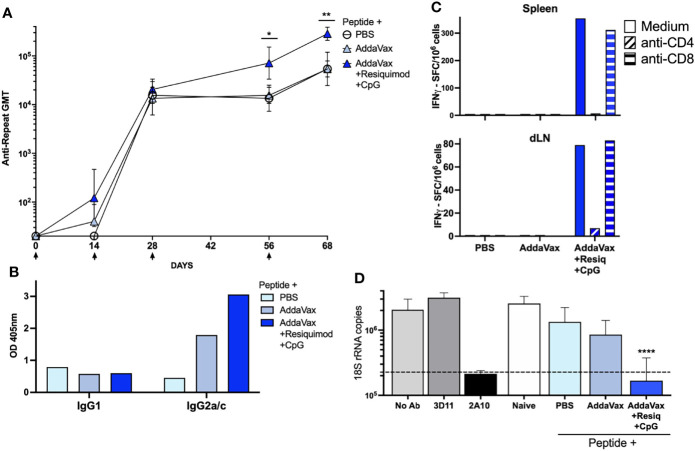
SS with *P. falciparum* CS peptide in AddaVax containing TLR agonists elicits Th1- type neutralizing antibody and cellular responses. **(A)** Kinetics of anti-CS repeat IgG response following SS immunization. Sera was collected 14d post each SS immunization (arrows) with CS peptide in PBS, AddaVax or AddaVax + Resiquimod + CpG. ELISA geometric mean IgG titers (GMT) are shown for sera from individual mice (*n* = 5/group). Mann-Whitney test showed a significant difference in antibody titer post 3^rd^ dose (p = 0.0317) and post 4th dose (p = 0.0079) with SS immunization with CS peptide in AddaVax + Resiquimod + CpG compared to CS peptide in AddaVax only. Error bars show standard deviation. **(B)** Anti-CS repeat IgG isotypes in serum of SS immunized mice. ELISA was carried out using MAB specific for Th2-type IgG1 or Th1-type IgG2a/c. Data are shown as OD of pooled serum (1:320 dilution) obtained post fourth SS immunization from each group of mice (*n* = 5 mice/group). **(C)** T cell cytokine responses in spleen and dLN of SS immunized mice. IFNγ ELISPOT was carried out using pooled spleen or dLN cells (5 mice/group) obtained post fourth SS immunization with CS peptide in PBS, Addavax only or Addavax + Resiquimod + CpG. Data are shown as SFC/10^6^ after subtraction of medium only control. **(D)** Sporozoite neutralizing antibody in serum of SS hyperimmunized mice. TSNA was measured using serum (1:5 dilution) of individual mice (n = 5 mice/group) obtained post fourth SS immunization. Mean number of 18S rRNA copies in cultures were quantitated by qRT-PCR. Controls (gray bars) included MAB 2A10 as a inhibitory antibody positive control, or MAB 3D11 as a negative antibody control, and no antibody (No Ab). Dotted line indicates 90% reduction in parasite rRNA copy numbers. Serum from mice immunized SS with CS peptide in AddaVax + Resiquimod + CpG gave >90% inhibition (dotted line), with a significant reduction in parasite copy numbers when compared to naïve serum (One-way ANOVA of log transformed data with Dunnett’s multiple comparisons test and adjusted p values, ****p < 0.0001). No significant difference in 18S rRNA copy numbers was found in cultures containing serum of mice immunized SS with PBS or AddaVax compared to naïve. (*p = 0.0317) and (**p = 0.0079).

**Figure 2:**
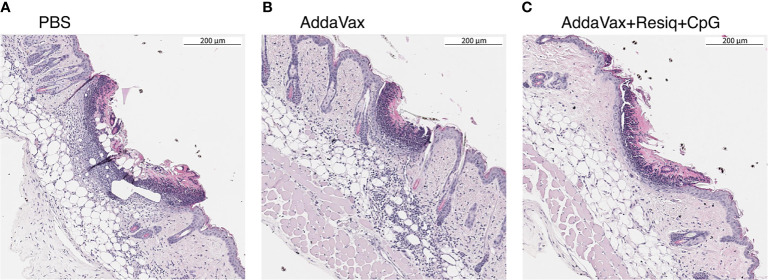
Cellular infiltration into dorsal skin 24h post SS prime. Nuclear staining with hematoxylin (H&E) was used as a measure of cellular infiltration. Dorsal skin sample were obtained at 24h post SS with CS peptide in **(A)** PBS, **(B)** Addavax, or **(C)** Addavax + Resiquimod + CpG. The pattern of intensity of cell infiltration, Addavax > PBS > AddaVax + Resiquimod + CpG was similar in dorsal skin from two independent experiments, with representative results of one experiment shown.

Mice from each experimental group (2 – 3 mice/group) were sacrificed at 4h or 24h post first SS immunization and 1cm^2^ skin samples were excised from the SS site and fixed in 4% paraformaldehyde. Samples were embedded in paraffin, sectioned and stained with H&E or labeled by immunohistochemistry (IHC) using 2 – 3 sections for each mouse (Histowiz Inc., NY). For IHC, fixed tissue sections were labeled with HRP-conjugated antibodies specific for murine CD11b+ monocytes (Abcam Cambridge, MA), F4/80+ macrophages (Invitrogen Thermo Fisher Scientific, Waltham MA), LY6G+ neutrophils (Abcam, Cambridge, MA), B220+ B cells (Novus Biologicals, Centennial, CO), or CD3+ and CD4+T cells (Abcam, Cambridge, MA). Cellular infiltration at the immunization site was examined in tissue sections from 2-3 mice/group and cellular density in the area under the SS scar was scored manually by two investigators using a range of 1+ to 4+ for intensity of cellular infiltration (**Figures 1S**, **2S**). Two independent experiments were carried out with similar results. Figures show results of a representative experiment.

### Cytokine/Chemokine Assays

Cytokines and chemokines were measured in serum collected 4 – 48h post SS priming and stored frozen at -80°C until tested. Samples were screened using a Cytokine/Chemokine Microarray (Cytokine array Q1, RayBiotech Inc, Norcross, GA) to measure IL-1β, IL-2,-3,-5,-6,-10, IL12p70, IL-13,-17,-17F,-21,-22,-23,-28, IFNγ, MIP-3α (CCL20), TGFβ1 and TNFα, according to the manufacturer’s protocol. Array slides were read with a 710AL scanner, using Mapix version 8.5.0 scanning software and results were analyzed by RayBiotech using Quantibody Q-Anlyzer software. Quantification of each cytokine/chemokine (pg/ml) was based on standards included in each microarray and the results expressed as fold-increase over limit of detection (LOD) with >3X LOD taken as positive. ELISA assays were used to measure IP-10 (CXCL10) (Abcam, Cambridge, MA) and IL-22 (R&D Systems, Minnesota, MN) in serum obtained at 4h and 24h post SS priming, according to the manufacturer’s protocol. All assays were repeated at least twice with results shown for representative experiment.

### Humoral immunity

IgG anti-CS repeat antibodies were measured in individual serum samples collected 14d post each SS immunization using a *P. falciparum* CS repeat peptide ELISA, as previously described (20, 29). Results are shown as geometric mean titers (GMT), with the endpoint defined as the final dilution giving an OD greater than three times the OD of BSA-coated control wells. Isotypes of anti-CS repeat IgG antibodies were determined by ELISA using MAB specific for murine Th2-associated IgG1 antibody or Th1-associated IgG2a/c antibody (Southern Biotechnology, Birmingham, AL).

The neutralizing activity of anti-repeat antibody elicited by SS immunization was measured using an *in vitro* Transgenic Sporozoite Neutralization Assay (TSNA), as in previous studies (20, 29, 30). Briefly, 2 X 10^4^ transgenic *P. berghei* sporozoites expressing the entire *P. falciparum* CS repeat region (termed PfPb) (31), were incubated with immune or naïve murine serum prior to addition to confluent cultures of human HepG2 hepatoma cells (ATCC HB 8065) in cRPMI (RPMI 1640 supplemented with 10% FBS, 50U Penicillin/50 µg Streptomycin, sodium pyruvate, non-essential amino acids, all from Gibco, ThermoFisher, Waltham, MA). Controls included sporozoites incubated with 25 µg/ml of monoclonal antibody (MAB) 2A10 (ATCC BEI MRA 183), specific for *P. falciparum* CS repeats, or MAB 3D11, specific for *P. berghei* CS repeats (9), as positive and negative controls, respectively. Plates were incubated at 5% CO_2_ for 48h, with media change at 24h, followed by extraction of total RNA (PureLink, RNA Mini Kit, ThermoFisher, Waltham, MA). The amounts of parasite 18S rRNA in each culture extract was quantitated by real-time PCR (qRT-PCR) using cDNA primers specific for 18S ribosomal RNA (rRNA) (30, 32). The parasite 18S rRNA copy number was calculated based on a standard curve generated with known amounts of plasmid 18S cDNA. As in previous studies (20), a >90% reduction in parasite copy number was considered biologically relevant as previous studies using injection of known numbers of sporozoites demonstrated that >90% reduction was associated with sterile immunity or delayed prepatent period (1, 33).

### Cellular Immunity

Th1- and Th2-type cells were measured in spleen cells obtained following the fourth SS immunization using IFNγ and IL-5 ELISPOT kits, respectively, per the manufacturer’s protocol (BD Biosciences, San Jose, CA). Pooled spleens (3-5 mice/group) were stimulated with CS peptide (10 µg/ml) or RPMI medium without peptide. PMA/Ionomycin stimulation served as positive control (Sigma, St. Louis, MO). Results were expressed as number of spot forming cells (SFC)/10^6^ spleen cells after subtraction of media control background. The phenotype of the cytokine-producing T cells measured in ELISPOT was determined by treatment with anti-CD4 (MAB GK1.5) or anti-CD8 (MAB 2.43) monoclonal antibodies (Bio X Cell, Lebanon, NH).

### Statistics

Statistical analysis was carried out using GraphPad Prism software version 9.2.0 (GraphPad Software, La Jolla, CA). For antibody analysis (**Figure 1A** and **Figure 4SB**), differences between groups were assessed by two-tailed, Mann-Whitney test. Comparison of IP-10 -/- to WT antibody titers (**Figure 6B**) was by one-way ANOVA with Bonferroni’s post-test for multiple comparisons. For TSNA, differences between experimental groups and naïve control were determined by one-way ANOVA with Dunnett’s post-test for multiple comparisons. For analysis of parasite 18S rRNA copy number measured in TSNA, the average copy number was calculated across each trial (performed in triplicate) within each group. A P value <0.05 was considered significant and adjusted P values for multiple comparisons are given.

## Results

### SS With CS Peptide in Adjuvant Containing TLR Agonists Elicits Sporozoite Neutralizing Antibody

SS immunization with CS peptide in AddaVax containing a combination of the TLR 7/8 agonist Resiquimod and the TLR 9 agonist CpG elicited enhanced anti-CS repeat antibody titers when compared to AddaVax without TLR agonists (**Figure 1A**, p = 0.0317 post 3^rd^ dose; p = 0.0079 post 4^th^ dose), consistent with our previous studies (20). The addition to Addavax of Resiquimod and CpG TLR agonists led to production of Th1-type IgG2a/c anti-repeat antibodies (**Figure 1B**). In contrast, SS with CS peptide in AddaVax without TLR agonists, or in PBS, elicited a Th-2 type IgG1 antibody and minimal IgG2a/c antibody.

Spleen cells and draining lymph node (dLN) cells of mice immunized with CS peptide in AddaVax + Resiquimod + CpG had predominantly IFNγ-producing CD4+ T cells (**Figure 1C**), consistent with the shift to IgG2a/c isotype of anti-repeat antibodies. In contrast, cells from mice immunized with CS peptide in AddaVax only, or PBS, did not have detectable IFNγ-producing CD4+ T cells. Minimal IL-5 producing T cells were detected by ELISPOT (data not shown).

When the neutralizing function of the anti-repeat antibodies elicited by SS was assayed by TSNA, only the serum of mice immunized with CS peptide in AddaVax + Resiquimod + CpG neutralized sporozoite infectivity and inhibited >90% of parasite growth *in vitro* (**Figure 1D**). There was a statistically significant reduction in parasite rRNA copy number in cell cultures receiving PfPb sporozoites incubated with immune serum from mice immunized with CS peptide in AddaVax + Resiquimod + CpG compared to serum of naïve mice (p = 0.0001). Reduction in parasite copy number was comparable to that obtained with protective MAB 2A10. No significant difference in rRNA copy number was found using serum of mice immunized with CS peptide in PBS or AddaVax compared to naïve serum.

### Presence of TLR Agonists Modulate Cellular Infiltration at Skin Scarification Site

To examine the innate responses associated with induction of Th1-type humoral immunity following SS, dorsal skin obtained from the SS site harvested 4h or 24h post initial SS was stained with H&E (**Figure 2**). At 4h, only minimal histological changes were noted, while at 24h active wound healing and re-epithelization were clear at the SS site (**Figure 1S**). Skin obtained at 24h post SS was therefore examined in more detail in the area under the SS scar (**Figure 2S**).

The intensity of the cellular influx at the SS site, reflected by nuclear (hematoxylin) staining in H&E sections, varied depending on the adjuvant formulation (**Figure 2**). When compared to immunization without adjuvant, SS with CS peptide in Addavax elicited a heavy cellular influx throughout the dermis and subdermis at the SS site (**Figure 2B** **)**. The lowest level of nuclear staining was in skin from mice immunized with CS peptide in AddaVax + Resiquimod + CpG **(** **Figure 2C** **)**. The intensity of cellular infiltration was AddaVax > PBS > AddaVax + Resiquimod +CpG at the SS site, with a similar pattern observed in two independent experiments.

**Figure 3:**
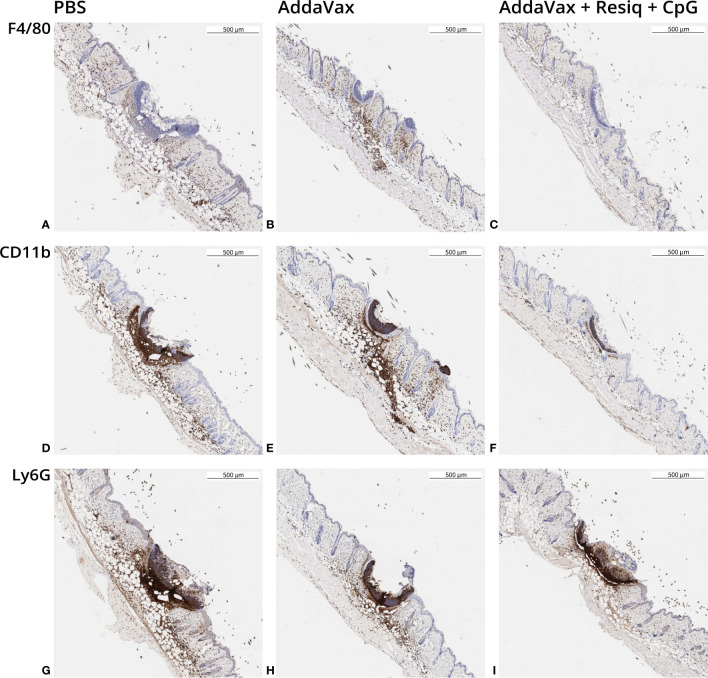
Immunohistochemistry (IHC) of dorsal skin obtained 24h post SS prime. Skin sections were obtained from 2-3 mice/experimental group at 24h post SS with CS peptide in PBS (first column), Addavax (second column) or Addavax + Resiquimod + CpG (third column). Cellular infiltration was assessed in skin sections from SS site labeled by IHC with antibody to F4/80 **(A–C)**, CD11b **(D–F)** or Ly6G **(G–I)**. Similar results were obtained in three experiments with results of a representative experiment shown.

Efforts to use flow cytometry to analyze the cell populations infiltrating the SS site at 24h was limited by technical difficulties in dissociating murine dorsal skin, as reported by others (34). Immunohistochemistry (IHC) of skin tissue sections was therefore used to examine the cell populations *in situ* at the SS site following labeling for monocytes (CD11b+), macrophages (F4/80+) and neutrophils (Ly6G+), as well as T (CD3+, CD4+) and B (B220+) cells (**Figure 3**).

SS with peptide in AddaVax led to a notable increase in F4/80+ cells at the SS site, when compared to SS without adjuvant (PBS) (**Figures 3A, B** **)**. The lowest F4/80 label intensity was observed following SS with AddaVax + Resiquimod + CpG (**Figure 3C** **)**. CD11b+ monocyte labeling (**Figures 3D, E** **)** and Ly6G+ neutrophil labeling (**Figures 3G, H** **)** was also strongest in skin of mice primed with peptide in AddaVax or PBS, with notably lower label intensity at the site following SS with AddaVax + Resiquimod + CpG **(** **Figures 3F, I** **).** The pattern of intensity of the IHC label was AddaVax > PBS > AddaVax + Resiquimod + CpG, consistent with the pattern observed in H&E stained sections of skin from the SS site **(** **Figure 2** **)**.

Immunization with or without adjuvant did not alter levels of T cells (CD3+) and B cells (B220+) detectable in skin sections by IHC at 24h post SS prime (data not shown). Only minimal numbers of B220+ B cells were observed scattered throughout the dermis at 24h post SS with or without adjuvant, similar to levels of B cells in naïve skin. CD3+ T cells were also not visibly increased post SS when compared to naïve mice, with the majority of T cells associated with hair follicles or scattered throughout the epidermis.

### Chemokines/Cytokines at 4h and 24h Post SS Prime

Cytokines and chemokines are required for repair of the skin barrier following trauma as well as to initiate adaptive immunity in response to skin infection or invasive pathogens. To examine the cytokines/chemokines that may have contributed to cellular responses noted at 24h, serum collected at 4h and 24h post prime was screened using a multiplex chemokine/cytokine microassay (RayBiotech Inc, Norcross, GA).

**Figure 4:**
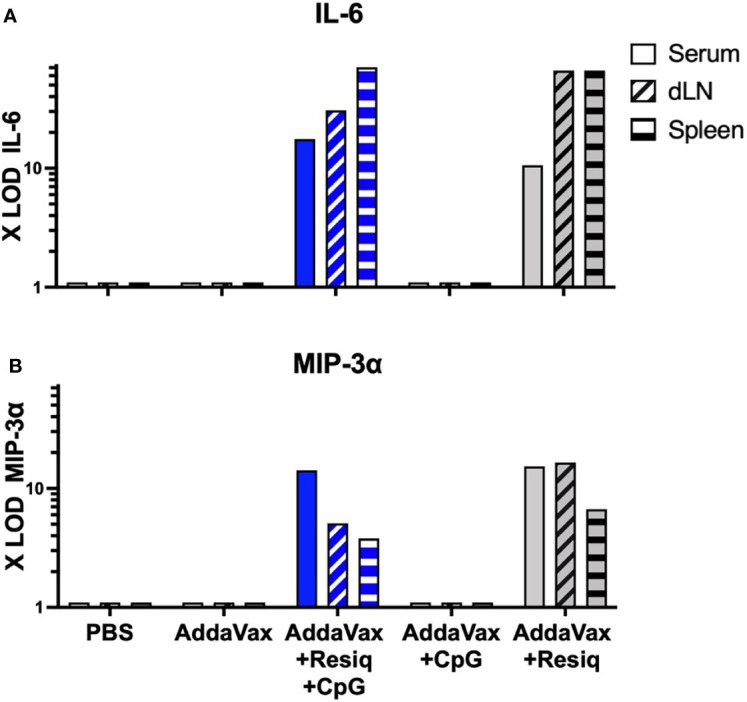
Cytokines/chemokines detectable by microarray at 4h post SS prime. Serum and extracts of spleen or dLN collected at 4h post SS from 3-4 mice/group were tested for levels of cytokines/chemokines by microarray. IL-6 **(A)** and MIP-3α **(B)** were detected in serum (solid blue bar) and extracts of spleen and dLN (hatched blue bars) obtained from mice immunized with AddaVax + Resiquimod + CpG, but not Addavax only or PBS. Induction of IL-6 and MIP-3α was primarily due to inclusion of Resiquimod (gray bars). All samples were negative (<3X LOD) for IL-1β, IL-2,-3,-5,-10, IL-12p70, IL-13,-17,-17F,-21,-23,-28, IFNγ, TGFβ1 and TNFα when tested by microarray.

As early as 4h post SS, elevated levels of pro-inflammatory cytokine IL-6 (**Figure 4A**) and chemokine MIP-3α (CXCL20) (**Figure 4B**) were detected in serum of mice immunized with peptide in AddaVax + Resiquimod + CpG **(** **Figure 4**, solid blue bars). Tissue extracts obtained from draining lymph node (dLN) and spleen at 4h post prime with CS peptide in Addavax + Resiquimod + CpG also had increased levels of IL-6 and MIP-3α (hatched blue bars). The elevated levels of cytokines/chemokines were elicited primarily by the TLR 7/8 agonist Resiquimod, as SS with AddaVax + CpG did not elicit detectable IL-6 or MIP-3α in serum or extracts of dLN and spleen (**Figures 4A, B**, gray bars). The increase in IL-6 and MIP-3α was transient, as sera collected at 24h post prime showed minimal IL-6 or MIP-3α signals (data not shown). Serum collected at 4h or 24h post prime with CS peptide in Addavax without TLR agonists, or peptide without adjuvant (PBS) did not have detectable levels of IL-6 or MIP-3α (<3X LOD). Tissue extracts from naïve mice were also negative at both time points (data not shown). All tissue and serum samples were negative for other cytokines/chemokines included in the microassay: IL-1β, IL-2,-3,-5,-10, IL12p70, IL-13,-17,-17F,-21,-22,-23,-28, IFNγ, TGFβ1 and TNFα.

The composition of the antigen used in SS could potentially contribute to the cytokine/chemokine pattern if the immunogen contained TLR ligands due to microbial contaminants. In the current study, the CS synthetic peptide did not contribute to the chemokine/cytokine responses measured at 4h, as IL-6 and MIP-3α were also detected in serum of mice immunized with AddaVax + Resiquimod + CpG without CS peptide (**Table 1S**). As found following SS with CS peptide with AddaVax + Resiquimod + CpG, the elevated cytokines/chemokine responses post immunization without peptide were transient. IL-6 levels decreased from 36.9X LOD at 4h to 8.9X LOD, while MIP-3α levels decreased from 319.7X LOD at 4h to 16X LOD at 24h post SS. Similar to SS with CS peptide, the elevated cytokines/chemokines were elicited primarily by the TLR 7/8 agonist Resiquimod, with only low levels of IL-6 (3.1 X LOD) and MIP-3α (26.3X LOD) detectable in serum following SS with AddaVax + CpG. The serum from mice immunized with AddaVax + Resiquimod + CpG, or with AddaVax + Resiquimod also had low levels of IL-12 and IL-22 detectable at 4h post prime. SS with AddaVax did not elicit IL-6, IL-12 or IL-22, while minimal levels of MIP-3α were detected at 4h and 24h. TNFα was only detected is serum following immunization without TLR agonists (PBS, AddaVax).

**Figure 5:**
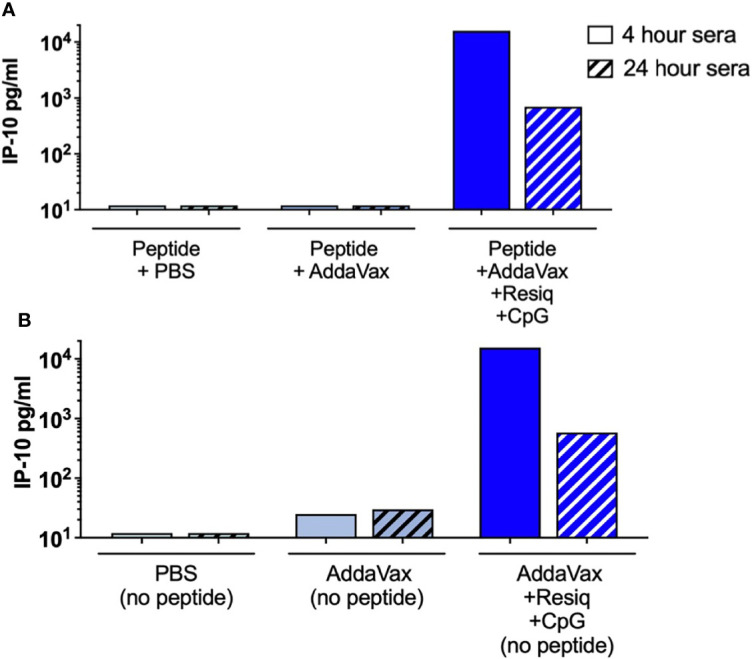
IP-10 (CXCL10) in serum 4h and 24h post SS with AddaVax + Resiquimod + CpG. ELISA quantitation of chemokine IP-10 (CXCL10) in serum of mice immunized by SS either with CS peptide **(A)** or without peptide **(B)**. IP-10 concentrations (pg/ml) were measured in serum collected at 4h (solid bars) or 24h (hatched bars) post SS prime.

While the microarray provided a significant advantage in screening for multiple cytokines/chemokines in single samples, the assay did not include chemokines such as IP-10 (CXCL10) which is known to play a role in the initiation of Th1-type adaptive immunity (35). An ELISA was therefore used to measure IP-10 in serum collected at 4h and 24h post SS prime. Elevated levels of IP-10 were detected in serum at 4h (18,690 IP-10 pg/mL, with decreased levels at 24h, post SS with peptide in AddaVax + Resiquimod + CpG (**Figure 5A** **).** A second experiment detected 15,911 IP-10 pg/mL confirming the elevated level of IP-10 at 4 hours post prime (data not shown). IP-10 was not detected in serum following SS with peptide in Addavax only, or with peptide in PBS, at either 4h or 24h post prime. Elevated IP-10 at 4h, with reduced levels at 24h, was also observed following SS with AddaVax + Resiquimod + CpG without peptide **(** **Figure 5B** **)**, indicating that the CS peptide did not contribute to the induction of the IP-10 chemokine, consistent with cytokines/chemokines detected by microarray. Additionally, SS using single TLR agonists demonstrated that IP-10 was elicited primarily by the formulation containing Resiquimod, with little or no IP-10 detected in the AddaVax + CpG group (data not shown).

### Innate and Adaptive Immune Responses in IP-10 -/- Mice

The pro-inflammatory cytokines/chemokines reproducibly detected at 4h post prime, IL-6, MIP-3α, IL-22 and IP-10, have pleomorphic functions in tissue regeneration, inflammation, as well as initiation and modulation of adaptive immune responses. To begin to explore the roles of the complex array of cytokines/chemokines elicited by SS with AddaVax + Resiquimod + CpG in the adaptive immune response, we focused on IP-10 (CXCL10). Previous studies in non-human primates (NHP) had shown that the intradermal injection of adjuvant containing Resiquimod + CpG led to detectable IP-10 in serum at 3h, and increased inflammatory monocytes in dLN at 24h (36). IP-10 was also the most consistently elevated early chemokine detected in NHP plasma at 6h - 24h post priming with HIV gp140 in an oil adjuvant containing Resiquimod + CpG and was associated with strong antibody responses following multiple IM injections (37).

To examine the role of IP-10 in innate and adaptive immune responses following immunization, mice lacking IP-10 (IP-10 -/-) were immunized with CS peptide in Addavax with or without TLR agonists. When skin samples obtained at 24h post SS were labeled by IHC, the IP-10 -/- mice immunized SS with AddaVax demonstrated strong F4/80+ and Ly6G+ cellular infiltration, similar to WT controls (**Table 1**). Similar to WT, skin from the IP-10 -/- mice immunized with AddaVax + Resiquimod + CpG had reduced F4/80 and Ly6G labeling when compared to skin from IP-10 -/- mice immunized with CS peptide in AddaVax or PBS. However, in contrast to WT, the IP-10 -/- mice had increased CD11b+ cells following SS with AddaVax + Resiquimod + CpG.

**Table 1:** IHC of skin tissue from SS site of IP-10 -/- vs WT mice.

SS with CS peptide in		CD11b		F4/80		Ly6G	
		WT	IP-10 -/-	WT	IP-10 -/-	WT	IP-10 -/-
							
PBS		2.5	2.25	1.5	2.0	2.5	2.5
							
AddaVax		3.3	4.0	3.0	3.0	3.2	3.0
AddaVax	+ Resiquimod +CpG	1.9	3.25	1.25	1.0	2.0	1.25

IHC-labeled dorsal skin obtained 24h post SS prime was scored manually by microscopy: 1+, scattered positive cells; 2+, staining localized to SS site; 3+, increased cellular infiltration localized to SS; 4+, strong cell infiltration localized to SS. Skin sections from naïve WT or IP-10 -/- mice were scored 1+. Results are shown as average scores for slides from 2-3 mice/group. IHC using antibodies for CD3, CD4 and B220 scored 1+ for all experimental conditions (data not shown).

When serum of IP-10 -/- mice was assayed for cytokines/chemokines by microarray, elevated IL-6 and MIP-3α were detected in serum at 4h post prime with AddaVax + Resiquimod + CpG, similar to WT mice (**Table 2**). The concentrations of IL-6 and MIP-3α were 6-7 fold higher in the IP-10 -/- mice compared to WT mice. Low levels of IL-22 and TNFα were also detected in the serum of the IP-10 -/- mice following SS with Addavax + Resiquimod + CpG. A more sensitive IL-22 ELISA confirmed the presence of IL-22 in serum of both IL-10 -/- and WT mice at 4hrs post prime with peptide in AddaVax + Resiquimod + CpG (**Figure 3S**). Despite the enhanced sensitivity of the ELISA compared to microarray, minimal or no IL-22 was detected in serum of either IP-10 -/- or WT mice immunized SS with CS peptide in AddaVax or PBS.

**Table 2:** Chemokines/Cytokines in IP-10 -/- vs WT mice^a^.

Chemokine/Cytokine	LOD (pg/ml)	Serum obtained +4h post SS with CS peptide in					
		PBS		Addavax		AddaVax	
						+ Resiq + CpG	
** **		WT	IP-10 -/-	WT	IP-10 -/-	WT	IP-10 -/-
IL-6	19.6	<3X	<3X	<3X	<3X	17.6X	98.7X
IL-22	58.6	<3X	<3X	<3X	<3X	<3X	6.7X
MIP-3α	7.5	<3X	<3X	<3X	<3X	14.2X	104.9X
TNFα	31.8	<3X	<3X	<3X	<3X	<3X	6.8X

Chemokines/cytokines in serum obtained 4h post SS were quantitated by microarray. Results are shown as fold-increase over limit of detection (LOD), with <3X LOD considered negative. Serum from naïve WT or naïve IP-10 -/- mice were negative for all chemokines/cytokines (data not shown). Sera of IP-10 -/- and WT mice obtained 4h post SS prime with CS peptide with or without adjuvant were negative for: IL1-β, IL-2,-3,-5,-10, IL-12p70, IL-13,-21,-23,-28, IFNγ, and TGFβ1. IP-10 -/- mice immunized SS with AddaVax + Resiquimod + CpG had low levels of IL-17F (3.4X LOD) (not shown).

**Figure 6:**
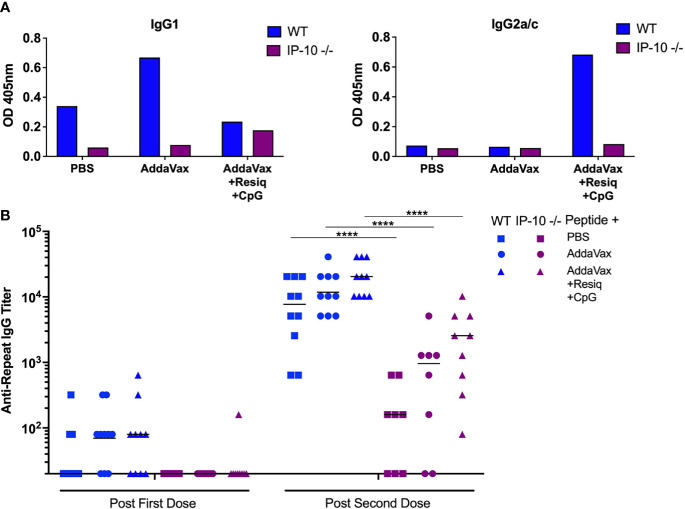
Anti-repeat antibody responses in IP-10 -/- mice post SS prime and boost. **(A)** IgG1 (left panel) and IgG2a/c (right panel) anti-CS repeat antibodies were measured by ELISA in pooled serum (3-4 mice/group) in IP-10 -/- mice (purple bars) or WT controls (blue bars). Serum was collected following SS prime and boost with CS peptide in PBS, Addavax or Addavax + Resiquimod + CpG. Results are shown as OD at 1:80 serum dilution. **(B)** Anti-repeat antibody response in serum of individual IP-10 -/- mice (purple symbols) compared to WT mice (blue symbols) following SS prime and boost with CS peptide without adjuvant (PBS), with AddaVax, or with AddaVax + Resiquimod + CpG. Results of two experiments were pooled with individual mice shown as data points and geometric mean indicated by bar. One-way ANOVA of log-transformed values followed by Bonferroni’s multiple comparisons test with adjusted p values, ****p <0.0001.

The adaptive immune response in IP-10 -/- mice was assayed by measuring IgG anti-repeat antibody following SS priming and boost. Previous studies in IP-10 -/- mice had demonstrated reduced primary IgG2a/c antibody responses following i.p. immunization with ovalbumin in Freund’s adjuvant (38). Following SS with peptide in AddaVax + Resiquimod + CpG, the IP-10 -/- mice had lower Th1-type anti-repeat IgG2a/c antibody when compared to WT mice (**Figure 6A**, right panel). In contrast, following SS with AddaVax + Resiquimod + CpG, the Th-2 type IgG1 response was similar in WT as compared to IP-10 -/- mice (left panel).

The primary anti-repeat antibody titers in the IP-10 -/- mice immunized SS with CS peptide in AddaVax + Resiquimod + CpG were significantly reduced when compared to WT mice (**Figure 6B**, p<0.0001). The reduced IgG2a/c concentrations and lower antirepeat antibody titers in the IP-10 -/- mice suggest that IP-10 plays a critical role in the shift to the Th1-type IgG subtype as well as the kinetics of the primary anti-repeat antibody response following SS immunization.

**Figure 7:**
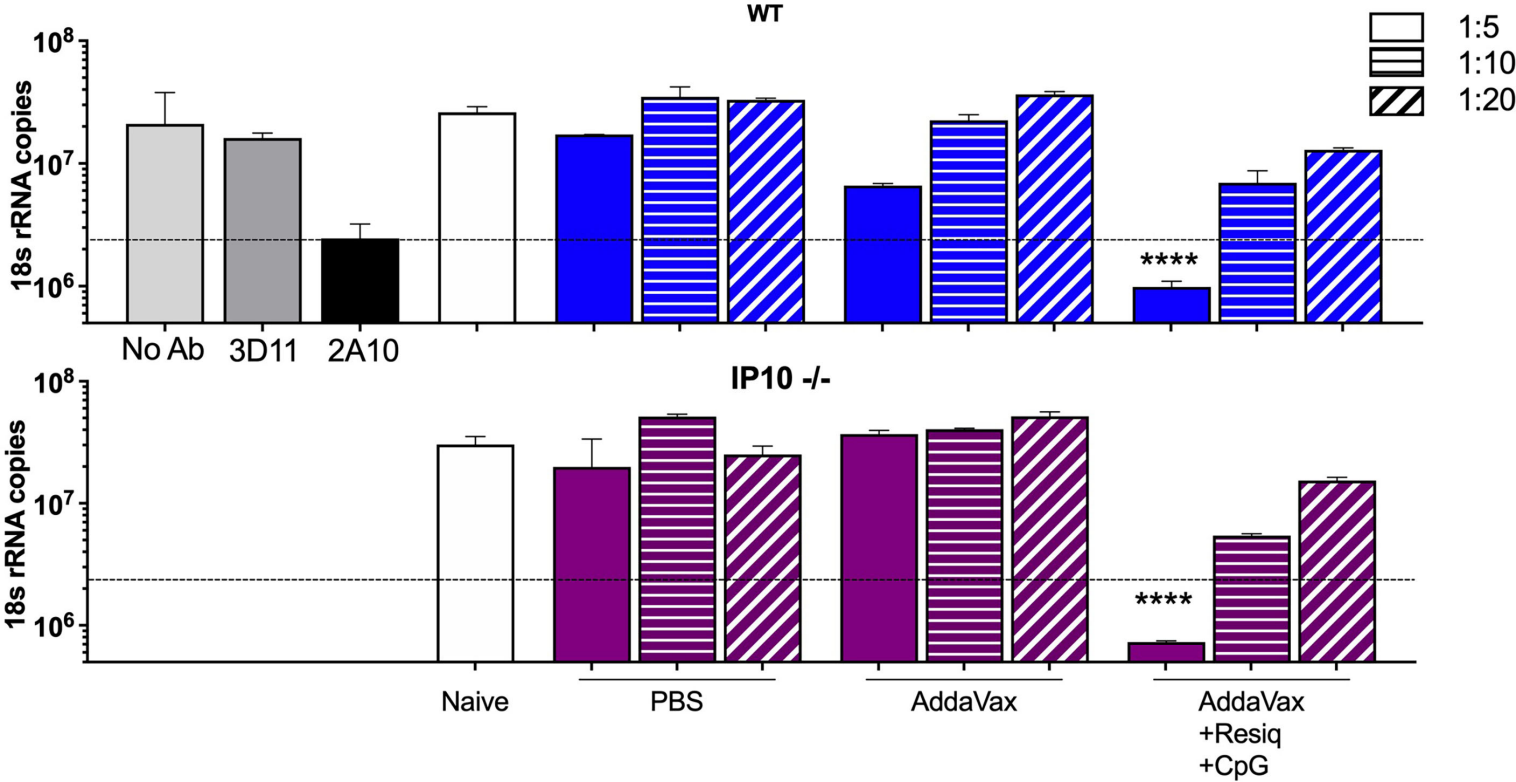
Sporozoite neutralizing antibody in hyperimmune serum of SS immunized IP-10 -/- compared to WT mice. TSNA was carried out with pooled immune sera (5 mice/group) obtained post 4^th^ SS immunization from WT (upper panel, blue bars) or IP-10 -/- mice (purple bars) tested in parallel at 1:5 –1:20 serum dilution. Controls (grey bars) include PfPb sporozoites incubated with inhibitory MAB 2A10 or with negative control MAB 3D11 or without antibody (No Ab). Dotted line represents >90% inhibition of parasite levels in liver cell cultures measured by qRT-PCR. Significant reduction of parasite copy number was obtained with hyperimmune serum from both IP-10 -/- mice and WT mice following four SS immunizations with peptide in AddaVax + Resiquimod + CpG compared to serum from naïve mice (one-way ANOVA of log-transformed data with Dunnett’s multiple comparisons test and adjusted p values, ****p <0.0001).

To investigate the function of antibodies elicited in the absence of IP-10, the IP-10 -/- mice received two additional boosters for a total of four SS immunizations (hyperimmunized), as our previous studies had found that development of neutralizing antibodies requires multiple boosters (20). Following a total of four immunizations with CS peptide in AddaVax + Resiquimod + CpG, hyperimmunized IP-10 -/- mice demonstrated a shift to IgG2a/c (**Figure 4SA**) and kinetics of anti-repeat antibody response **(** **Figure 4SB**) similar to that observed in WT mice (**Figure 1**). When hyperimmune serum of IP-10 -/- and WT mice were tested in parallel in TSNA, comparable neutralizing antibody levels were obtained in serum from IP-10 -/- mice and WT mice **(** **Figure 7** **)**. A 1:5 dilution of hyperimmune serum of both IP-10 -/- and WT mice immunized SS with AddaVax + Resiquimod + CpG reduced parasite levels *in vitro >*90%. A statistically significant reduction in rRNA copy number was found in cell cultures containing hyperimmune serum of IP10 -/- or WT mice when compared to serum from naïve mice (p<0.0001). No significant difference in parasite levels was found in cultures receiving sporozoities incubated in serum of IP-10 -/- or WT mice immunized with peptide in AddaVax or PBS.

## Discussion

Skin scarification (SS) with a *P. falciparum* CS repeat peptide delivered in an adjuvant formulation comprised of AddaVax containing a combination of the TLR 7/8 agonist Resiquimod and TLR 9 agonist CpG elicited anti-repeat antibodies that neutralized sporozoite infectivity (**Figure 1**), confirming our previous study (20). As early as 24h post prime, dorsal skin from the SS site, stained by H&E or labeled by IHC, demonstrated that the innate immune responses differed depending on the presence or absence of TLR agonists (**Figures 2**, **3**). Of note was the reduced cellular infiltration in the skin following treatment with AddaVax + TLR agonists (**Figure 2C**, **Figures 3C, F, I**) when compared to AddaVax only. Previous murine studies using a skin laser adjuvant, also noted a reduced cellular infiltrate following topical treatment with a TLR 7 agonist which the authors demonstrated was due to increased rapid transit of APC from the skin to the dLN (39).

The reduced cellular infiltration observed in the skin at 24h post SS with AddaVax + Resiquimod + CpG correlated with the presence of pro-inflammatory cytokines/chemokines, including IL-6, IL-22, MIP-3α (CCL20) and IP-10 (CXCL10), detectable at 4h post prime in serum as well as in extracts of dLN and spleen (**Figures 4**, **5**). These pro-inflammatory cytokines/chemokines were not detected in serum obtained 4h or 24h post SS with either AddaVax or PBS.

In this initial exploration of the potential role of the pro-inflammatory cytokines/chemokines detected at 4h in the induction of the adaptive immune response, we focused on IP-10 (CXCL10). IP-10 and the CXCR3 receptor in dLN are known to be of importance in the interaction of CD4+ T cells and dendritic cells and localization of Th cells to B cell areas to facilitate antibody isotype switching and affinity maturation (35, 40). Direct administration of IP-10 as adjuvant for peptide or protein antigens has been shown to elicit murine Th1-type antibody and CD8+ T cells (41). In addition to murine studies, intradermal injection of Resiquimod or CpG into Rhesus monkeys elicited IP-10 in serum detected 3-8h post injection and increased expression of IP-10 mRNA in LN (36). When serum of NHP immunized IM with HIV gp140 in an oil emulsion containing Resiquimod + CpG was tested using a panel of 30 cytokines/chemokines, only elevated serum IP-10 at 24h post prime correlated with enhanced anti-HIV antibody responses following additional boosters (37).

In the current studies, IP-10 -/- mice had reduced Th1-type IgG2a/c antibody and significantly lower anti-repeat titers following SS prime and boost with CS peptide in AddaVax + Resiquimod + CpG (**Figure 6**). These results are consistent with previous studies in IP-10 -/- mice that found impaired CD4+ Th1 cell responses and decreased IgG2a/c antibody titers following priming with soluble antigen (38). In the current studies, serum of IP-10 -/- mice had levels of IL-6, IL-22 and MIP-3α equal to or greater than WT mice at 4h post SS with AddaVax + Resiquimod + CpG (**Table 2**, **Figure 3S**), suggesting that these pro-inflammatory cytokines/chemokines were not sufficient to overcome the lack of IP-10 in the initiation of IgG2a/c antibody response.

In the IP-10 -/- mice, IHC labeling of skin sections obtained 24h following SS with AddaVax + Resiquimod + CpG demonstrated that CD11b+ cells were increased in IP-10 -/- mice compared to WT (**Table 1**). In contrast, the pattern of infiltration with F4/80+ and Ly6G+ cells was reduced, similar to the pattern observed in WT mice. CD11b+ antigen presenting cells (APC) are important in the transport of antigen from the injection site to the dLN. Following sporozoite injection ID or by mosquito bite, intravital microscopy of ear pinnae demonstrated increased numbers of CD11b+ cells at 2-4h and sporozoites associated with CD11b+ cells in dLN (42, 43). While the role of CD11b+ cells as APC following SS remains to be explored, the increase of CD11b+ cells at the SS site in IP-10 -/- mice immunized with AddaVax + Resiquimod + CpG could reflect a failure or delay of APC to transit out of skin to the dLN in the absence of an IP-10 signal. The resulting reduced APC:T cell interaction and Th1 differentiation in the dLN could potentially lead to reduced levels of anti-repeat IgG2a/c, as found in the IP-10 -/- mice following prime and boost with AddVax + TLR agonists (**Figure 6**).

Of note was the finding that multiple SS immunizations could overcome the reduced primary anti-repeat antibody found in the IP-10 -/- mice. Following four immunizations with AddaVax + Resiquimod + CpG, the skewing to IgG2a/c isotype and the magnitude of anti-repeat and neutralizing antibodies in serum of hyperimmunized IP-10 -/- mice were comparable to WT mice (**Figure 4S**, **Figure 7**). These finding suggest compensatory pro-inflammatory chemokines may be functioning in anti-repeat antibody response in IP-10 -/- mice following multiple SS immunizations. MIG (CXCL9) is also an agonist for the CXCR3 receptor which can function in differentiation of Th1 cells and antibody responses (35, 44). Whether CXCL9 can compensate for the lack of IP-10 in the hyperimmunized IP-10 -/- mice remains to be explored.

The reduced primary antibody responses in the SS primed IP-10 -/- mice suggest that IP-10 may provide a potential early biomarker for the initiation of Th1-type anti-repeat antibody response. Consistent with the murine studies, in NHP the presence of IP-10 following priming with HIV antigen in an oil adjuvant containing Resiquimod + CpG correlated with subsequent antibody development following five IM immunizations (37). It is encouraging that despite the variation in TLR distribution in mice versus NHP, the murine innate immune responses observed in the current studies were consistent with TLR stimulated antibody responses in NHP (45, 46). Measurement of IP-10 may therefore provide a useful early serologic marker for rapid screening of TLR agonist based adjuvants not only in murine, but also in primate hosts.

The ability to rapidly and easily test multiple iterations of TLR agonist concentrations and combinations is critical for the successful optimization of vaccine formulations for delivery to the skin. Skin delivery of vaccines provide the advantage of simplifying vaccination strategies and reducing costs by eliminating the need for trained medical personnel required for sterile injections. Co-delivery of antigen in TLR agonist adjuvants to skin APC by SS may more accurately mimic delivery of sporozoite and pathogen associated molecular patterns (PAMP) into the skin. The current studies demonstrate that a simple bifurcated needle can provide an inexpensive tool to deliver subunit vaccines to the skin. Further modifications of vaccine formulations by conjugation of TLR agonist and antigen may enhance vaccine potency (23, 42). Moreover, the use of more technologically advanced skin delivery systems, such as patches comprised of microneedles (47), would be expected to further improve vaccine immunogenicity by increasing the dose of antigen and/or TLR agonists delivered to the skin. Phase I trials of flu vaccine delivered to skin *via* a microneedle patch demonstrated that self-administered vaccines can elicit virus neutralizing antibody titers similar to IM immunization by trained healthcare personnel (48). Measurement of IP-10 in serum may facilitate rapid testing of various TLR agonist combinations and delivery systems, to determine whether these modifications lead to increased levels of IP-10 that correlate with increases in vaccine efficacy.

## Data Availability Statement

The raw data supporting the conclusions of this article will be made available by the authors, without undue reservation.

## Ethics Statement

The animal study was reviewed and approved by Institutional Animal Care and Use Committee, NYU School of Medicine.

## Author Contributions

EN, RM, and UF contributed to the design of the study. RM, RJ, SG, and RA performed the experiments. EN and RM wrote the manuscript. All authors reviewed the manuscript.

## Funding

We are grateful for the research support provided by NIH grant AI098302.

## Conflict of Interest

The authors declare that the research was conducted in the absence of any commercial or financial relationships that could be construed as a potential conflict of interest.

## Publisher’s Note

All claims expressed in this article are solely those of the authors and do not necessarily represent those of their affiliated organizations, or those of the publisher, the editors and the reviewers. Any product that may be evaluated in this article, or claim that may be made by its manufacturer, is not guaranteed or endorsed by the publisher.

## Supplementary Material

Supplementary Figure 1 H&E staining of dorsal skin obtained post SS prime with CS peptide in PBS showing the epidermal, dermal and subdermal skin layers. **(A)** At 4h, the skin exhibited minimal histological changes post SS regardless of adjuvant formulation. **(B)** At 24h, the SS site showed wound repair and re-epithelialization in all experimental groups.

Supplementary Figure 2 Scoring of IHC-labeled skin obtained 24h post SS prime. Following IHC labeling of skin, the area under the SS site (outline) was examined by microscopy for cellular infiltration. The intensity of cellular infiltration was scored as: 1+, scattered positive cells; 2+, cellular staining localized to SS site; 3+, increased cellular infiltration localized to SS site; 4+ heavy cellular infiltration at SS site.

Supplementary Figure 3 IL-22 in serum of IP-10 -/- and WT mice post SS prime. ELISA quantitation of IL-22 (pg/ml) in serum of IP-10 -/- (purple bars) as compared to WT mice (blue bars) at 4h post SS with CS peptide in PBS, Addavax, or Addavax + Resiquimod + CpG.

Supplementary Figure 4 Anti-repeat antibody response in hyperimmunized IP-10 -/- mice. **(A)** IgG isotypes measured in CS repeat peptide ELISA using pooled hyperimmune serum (1:5120 dilution) from IP-10 -/- mice obtained 14d post the fourth SS immunization. **(B)** Kinetics of anti-CS repeat IgG antibody measured by ELISA in serum of IP-10 -/- mice collected at 14d post each of four SS immunizations (arrows). Significant difference was found after SS immunization with CS peptide in AddaVax + Resiquimod + CpG compared to AddaVax only by Mann-Whitney test post 2nd dose (*p=0.0238), post 3rd dose (**p=0.0079), and post 4th dose (**p=0.0079).

## Abbreviations

CS, circumsporozoite protein; dLN, draining lymph node; IP-10, interferon gamma induced protein 10 (CXCL-10); MIG, monokine induced by gamma interferon (CXCL9); MIP-3α, MacrophageInflammatory Protein-3 alpha (CCL20); CXCR3, receptor for chemokines containing CXC conserved cysteine motif; MAB, monoclonal antibody; PfPb, transgenic *P. berghei* sporozoites expressing *P. falciparum* CS repeats; SS, skin scarification; TLR, Toll-Like Receptor; TSNA, Transgenic Sporozoite Neutralizing Assay.
