# Supplementary figures and images for: Innate Immune Responses and P. falciparum CS Repeat-Specific Neutralizing Antibodies Following Vaccination by Skin Scarification
Source: Front Immunol. 2022 Jun 6;13:801111. doi: 10.3389/fimmu.2022.801111 (PMC9207416; doi:10.3389/fimmu.2022.801111)

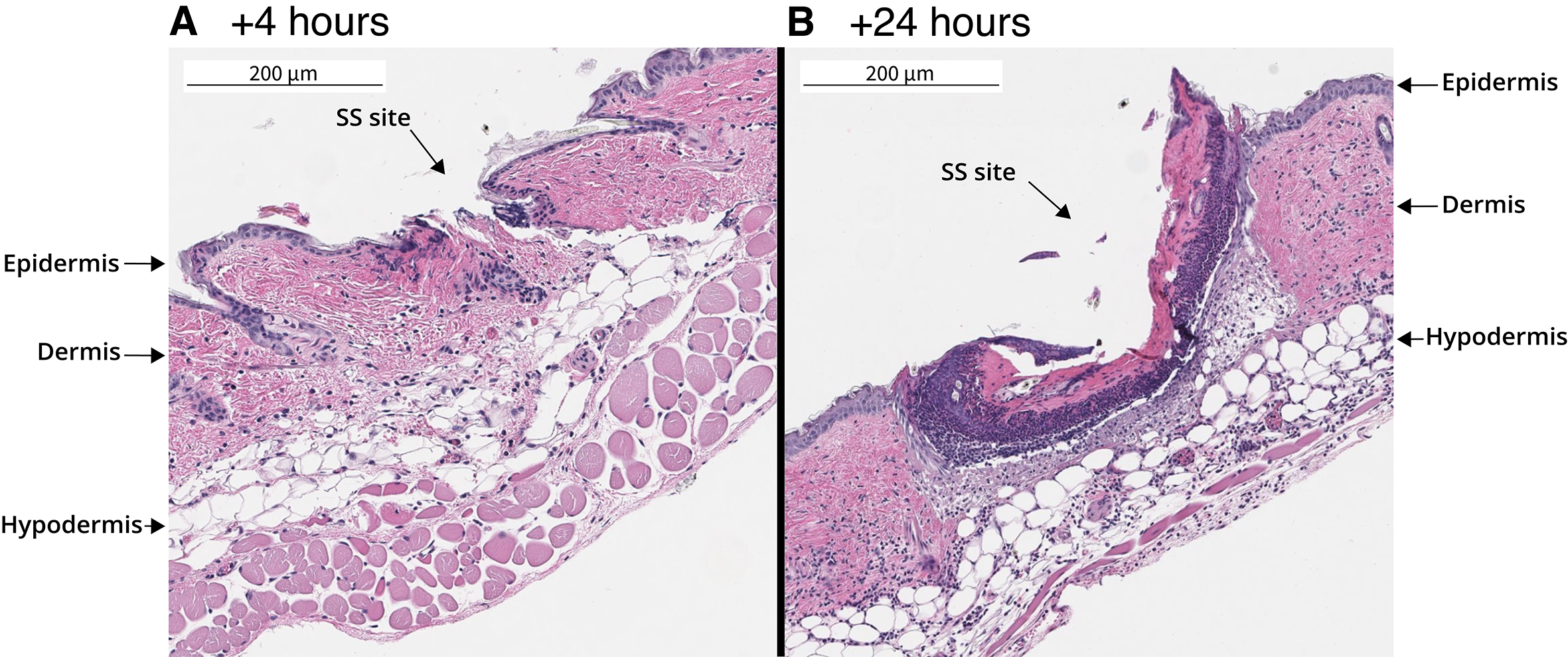

Supplement: Supplementary Figure 1 — H&E staining of dorsal skin obtained post SS prime with CS peptide in PBS showing the epidermal, dermal and subdermal skin layers. (A) At 4h, the skin exhibited minimal histological changes post SS regardless of adjuvant formulation. (B) At 24h, the SS site showed wound repair and re-epithelialization in all experimental groups. [file Image_1.tif]

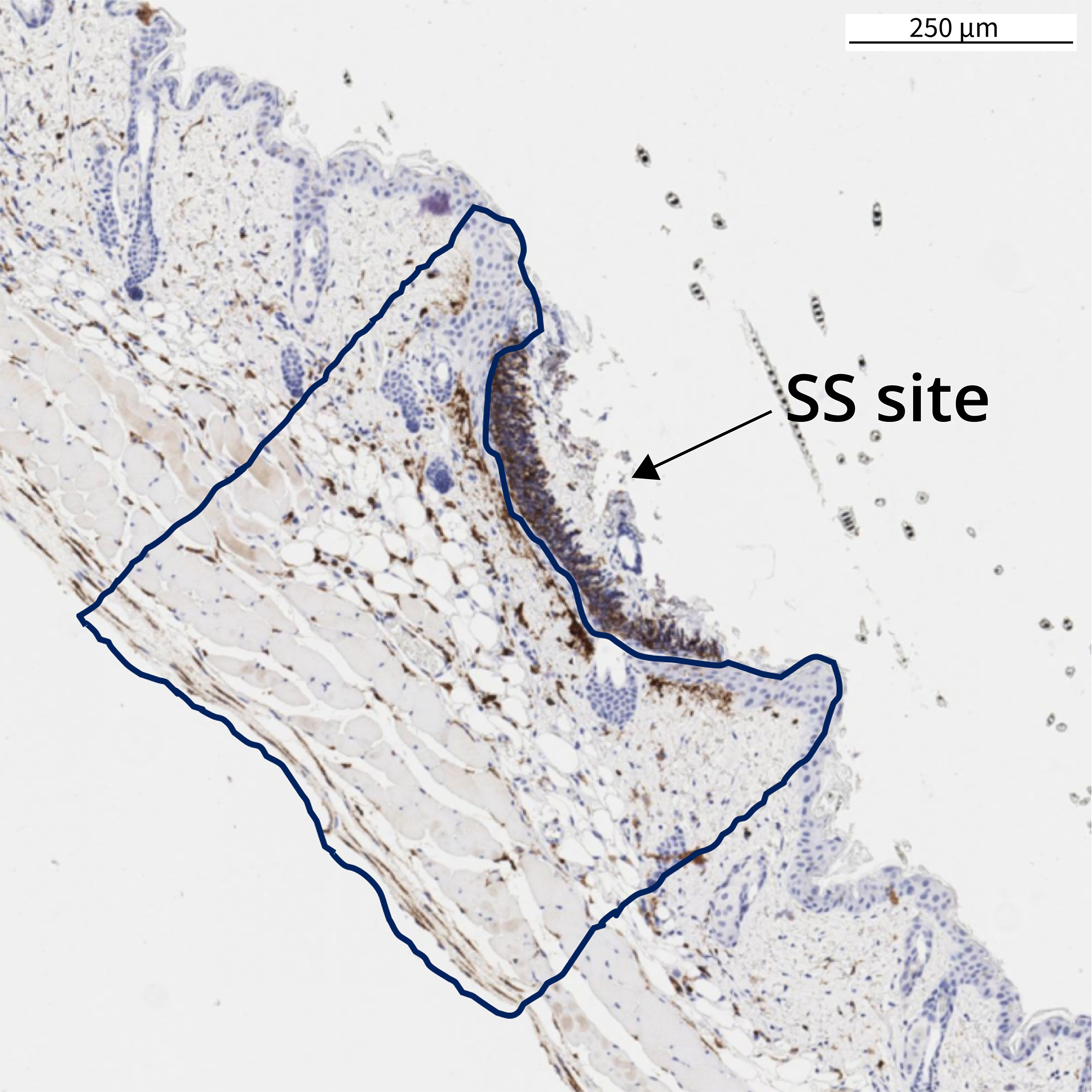

Supplement: Supplementary Figure 2 — Scoring of IHC-labeled skin obtained 24h post SS prime. Following IHC labeling of skin, the area under the SS site (outline) was examined by microscopy for cellular infiltration. The intensity of cellular infiltration was scored as: 1+, scattered positive cells; 2+, cellular staining localized to SS site; 3+, increased cellular infiltration localized to SS site; 4+ heavy cellular infiltration at SS site. [file Image_2.tif]

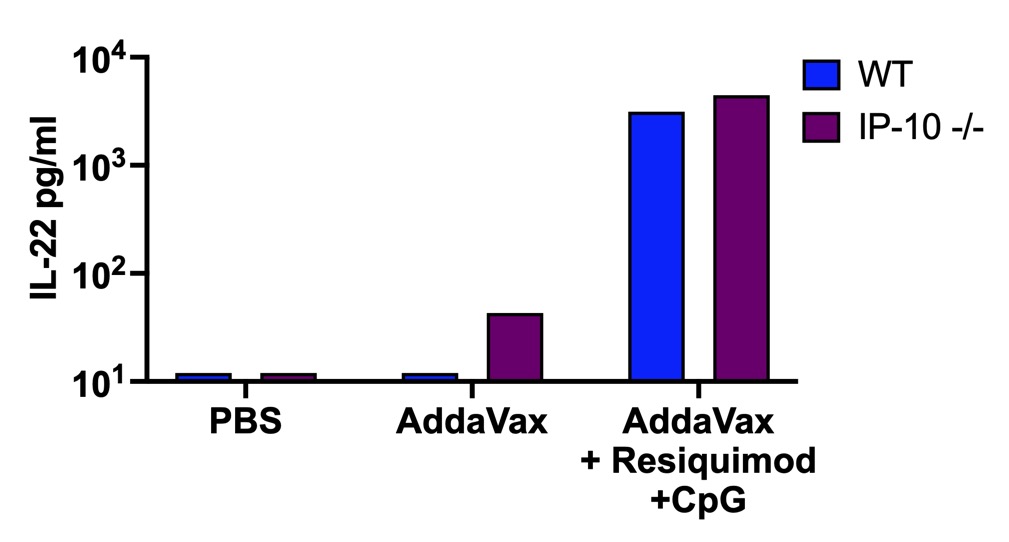

Supplement: Supplementary Figure 3 — IL-22 in serum of IP-10 -/- and WT mice post SS prime. ELISA quantitation of IL-22 (pg/ml) in serum of IP-10 -/- (purple bars) as compared to WT mice (blue bars) at 4h post SS with CS peptide in PBS, Addavax, or Addavax + Resiquimod + CpG. [file Image_3.jpeg]

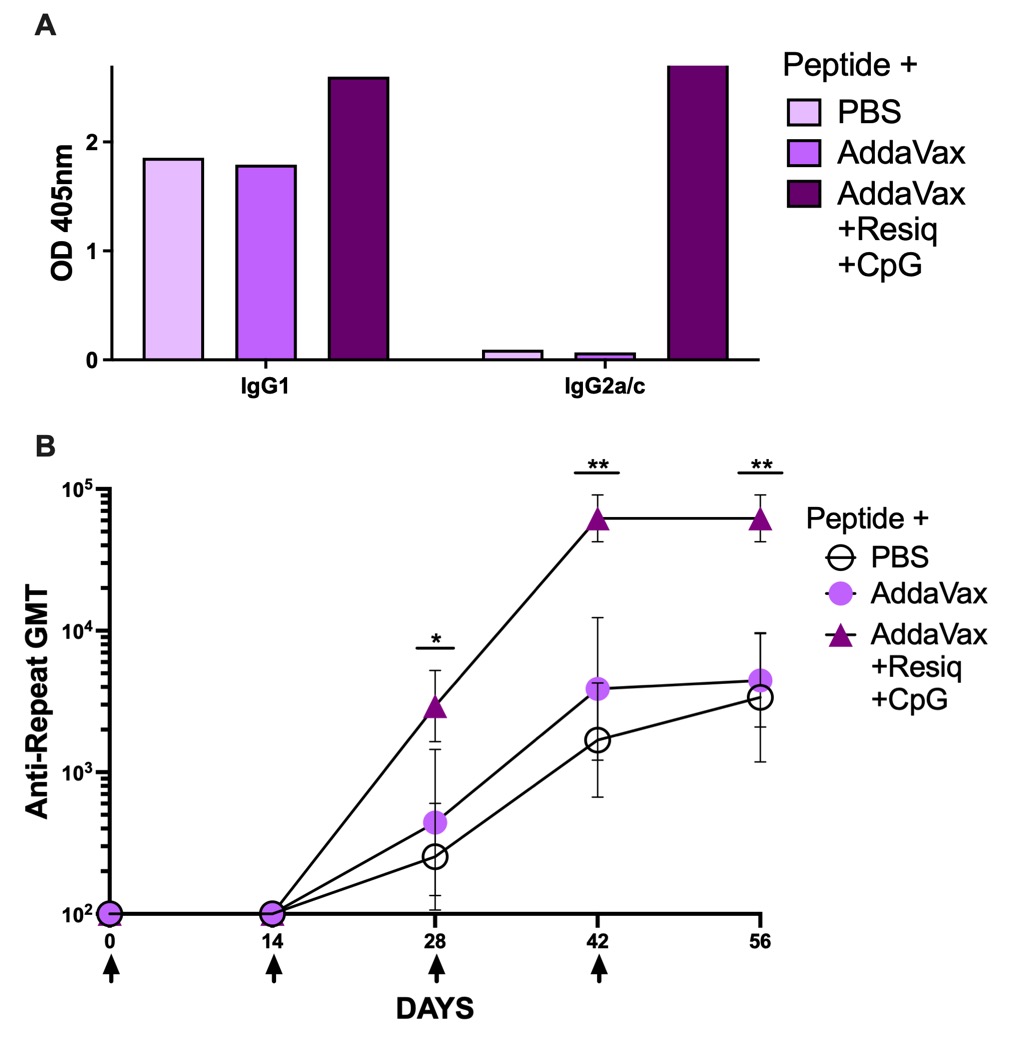

Supplement: Supplementary Figure 4 — Anti-repeat antibody response in hyperimmunized IP-10 -/- mice. (A) IgG isotypes measured in CS repeat peptide ELISA using pooled hyperimmune serum (1:5120 dilution) from IP-10 -/- mice obtained 14d post the fourth SS immunization. (B) Kinetics of anti-CS repeat IgG antibody measured by ELISA in serum of IP-10 -/- mice collected at 14d post each of four SS immunizations (arrows). Significant difference was found after SS immunization with CS peptide in AddaVax + Resiquimod + CpG compared to AddaVax only by Mann-Whitney test post 2nd dose (*p=0.0238), post 3rd dose (**p=0.0079), and post 4th dose (**p=0.0079). [file Image_4.jpeg]
