# Supplementary material for: Innate Immune Responses and P. falciparum CS Repeat-Specific Neutralizing Antibodies Following Vaccination by Skin Scarification
Source: Front Immunol. 2022 Jun 6;13:801111. doi: 10.3389/fimmu.2022.801111 (PMC9207416; doi:10.3389/fimmu.2022.801111)
Supplement: Supplementary file 5 [file Table_1.pdf]

Table 1S. Chemokines/cytokines in serum following SS without CS peptide

		PBS	AddaVax		AddaV + Resiq +CPG		AddaV + Resiq	AddaV + CpG
	LOD pg/ml	4 h	4 h	24 h	4 h	24 h	4 h	4 h
**IL-6**	20.2	**<**3X	<3X	<3X	36.9X	8.9X	84.5X	3.1X
**IL-12p70**	33.4	<3X	<3X	<3X	4.1X	<3X	12.9X	<3X
**IL-22**	72.0	<3X	<3X	<3X	6.6X	<3X	18.1X	<3X
**MIP-3α**	7.2	<3X	5.3X	3.9X	319.7X	16.0X	274.8X	26.3X
**TNFα**	27.7	13.8X	<3X	7.7X	<3X	<3X	<3X	<3X

Pooled sera from 3-4 mice/group obtained at 4h and 24h post SS prime were assayed by microarray. Results are shown as fold-increase over LOD (pg/ml), with 3X LOD taken as positive. Serum from naïve mice did not have detectable cytokines/chemokines (<3X LOD). Increase in: IL-2, IL-5, IL-10, IL-17, IL-23 and IL-28 were similar in all SS groups regardless of presence or absence of adjuvant (data not shown). No Increase in IL-1b, IL-4, IL-13, IL-17F, IL-21 was detected in any SS primed group.
